# Craniofacial Ossifying Fibromas in Children: Clinical Variability and Surgical Outcomes in a Case Series

**DOI:** 10.7759/cureus.102062

**Published:** 2026-01-22

**Authors:** Jocelyne García-Vela, Hiram H Plata-Huerta, Josefina Alejandra Morales Del Angel, Dariana Rodriguez

**Affiliations:** 1 Otolaryngology - Head and Neck Surgery, Universidad Autonoma de Nuevo Leon, Facultad de Medicina y Hospital Universitario "Dr. José Eleuterio González", Monterrey, MEX; 2 Otolaryngology - Head and Neck Surgery, Universidad Autonoma De Nuevo Leon, Facultad de Medicina y Hospital Universitario "Dr. Jose Eleuterio Gonzalez", Monterrey, MEX; 3 Otolaryngology - Head and Neck Surgery, Universidad Autonoma de Nuevo Leon, Facultad de Medicina y Hospital Universitario "Dr. Jose Eleuterio Gonzalez", Monterrey, MEX

**Keywords:** craniofacial tumors, juvenile ossifying fibroma, pediatric fibro-osseous lesions, pediatric sinonasal tumor, sinonasal tumors

## Abstract

Juvenile ossifying fibroma (JOF) is a rare benign fibro-osseous tumor affecting the craniofacial skeleton in children and adolescents. Although nonmalignant, it may behave aggressively and extend into adjacent structures such as the orbit or anterior skull base. Early symptoms are often subtle, delaying diagnosis. Two histologic variants exist - trabecular and psammomatoid - each with distinct clinical behavior and recurrence potential. We describe a case series of three pediatric patients (an 8-year-old male, a 13-year-old female, and a 16-year-old male) diagnosed with craniofacial JOF between March 2022 and January 2025. Presenting symptoms included progressive facial deformity, nasal obstruction, and proptosis. Imaging demonstrated expansile, well-defined lesions involving the ethmoid, maxillary, frontal, and sphenoid sinuses, with orbital displacement in all cases and anterior cranial fossa extension in one. All patients underwent endoscopic tumor resection; two required combined external approaches (Caldwell-Luc and Lynch incision) to achieve complete access. Significant intraoperative bleeding occurred in two cases but was successfully controlled. No permanent visual or neurological deficits were observed. At the six-month follow-up, none showed radiologic recurrence. JOF in pediatric patients is rare and may exhibit locally aggressive behavior, posing diagnostic and surgical challenges. Radiologic and histopathologic evaluation are essential for accurate differentiation from other fibro-osseous lesions. Complete excision via endoscopic or combined approaches provides excellent visualization and low morbidity while preserving function. Early recognition and multidisciplinary management optimize outcomes. Long-term imaging surveillance remains crucial due to the risk of late recurrence.

## Introduction

Fibro-osseous lesions comprise a heterogeneous group of bone disorders in which normal bone is replaced by a fibrous stroma containing variable amounts of mineralized material. Over the past few decades, the classification of these lesions, including fibrous dysplasia, osteoma, and ossifying fibroma (OF), has been substantially refined to better reflect their biological behaviors and histopathological features [1,2]. Ossifying fibroma (OF) is an infrequent benign neoplasm of mesenchymal origin, constituting approximately 10% of benign maxillary tumors. Occurrences in the ethmoid, frontal sinuses, or orbital regions are particularly uncommon [3-6]. Despite its benign histology, OF may demonstrate locally aggressive growth, progressive expansion, and erosion of adjacent structures, making complete surgical excision the treatment of choice [1,3-5].

Clinically, OFs are often asymptomatic in the early stages, becoming evident only after reaching a considerable size, which causes facial deformity, nasal obstruction, proptosis, diplopia, epiphora, or headaches [3,5,7,8]. In advanced cases, complications may include mucocele formation, visual impairment, and intracranial extension [9,10]. Several histopathological variants have been described, including cement-ossifying fibroma (COF), juvenile ossifying fibroma (JOF), and psammomatoid (JPOF) or trabecular (JTOF) subtypes [2,11,12]. JOF typically presents before the age of 15 and tends to exhibit a more aggressive clinical course with higher recurrence rates compared to the conventional adult type [2,10,11]. Among its subtypes, JTOF primarily involves the maxilla, whereas JPOF preferentially affects the paranasal sinuses and orbit [2,7,12]. Recognizing these variants is crucial because their clinical behavior, likelihood of recurrence, and surgical management vary significantly.

Given the rarity and aggressive nature of juvenile ossifying fibroma, particularly when it involves the ethmoid sinus and orbit, reports describing its variable presentation and surgical management remain scarce. Notably, there is an absence of established endoscopic or combined surgical protocols. This case series aims to contribute to the existing literature by presenting three pediatric cases with distinct clinicopathological features and therapeutic approaches.

## Case presentation

This retrospective case series included three consecutive patients diagnosed with ossifying fibromas of the paranasal sinuses between March 2022 and January 2025. Patients received treatment at the tertiary University Otolaryngology and Head and Neck Surgery Center within Dr. Jose Eleuterio Gonzalez Hospital, where they underwent surgical resection. Data was collected from the patients' medical records stored in our department’s internal database, including imaging studies, nasal endoscopies, and histopathology reports. Postoperative follow-up ranged from three to six months and included nasal endoscopy and control imaging. No additional cases that met the inclusion criteria were identified during this period. Given the limited number of cases, only descriptive data were analyzed, and no statistical analysis was performed.

Case 1

**Figure 1:**
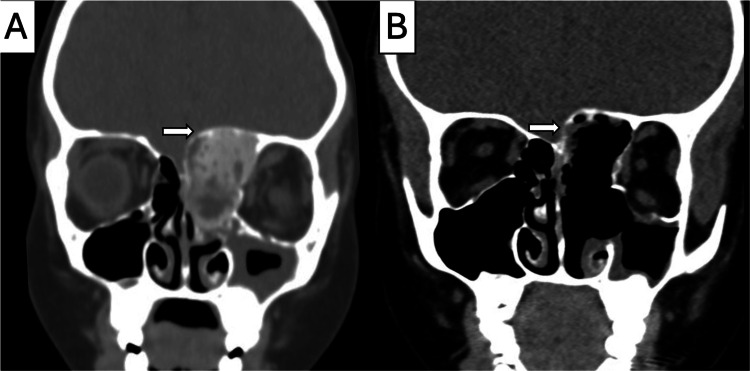
Case 1: Preoperative and postoperative CT. (A) Coronal view: a left ethmoidal tumour, heterogeneous with a central hypodense soft-tissue density, showing erosion of the lamina papyracea and anterior cranial base (white arrow). (B) Coronal view: post-surgical changes following maxillary antrostomy and left anterior ethmoidectomy; small remnants at the skull base were left in situ to avoid iatrogenic injury (white arrow).

An eight-year-old male with no significant past medical history presented with an 8-month history of progressive swelling of the left upper eyelid. Initial radiography revealed an orbital bone deformity. One week prior to admission, the patient underwent MRI and CT scans ordered by an ophthalmologist, which demonstrated an ossifying fibroma involving the left ethmoidal cells, causing deviation of the left globe with proptosis and extension into the left frontal sinus. The patient was subsequently referred to our institution for further evaluation and management. Clinical examination revealed left-sided proptosis without pain, fever, or visual impairment. Ocular mobility was also preserved. Nasal endoscopy revealed a rounded, smooth mass in the left nasal cavity that contacted the lateral nasal wall and medially displaced the middle turbinate. The patient underwent functional endoscopic sinus surgery. The mass was progressively resected, maxillary antrostomy was performed, the anterior and posterior ethmoidal cells were removed, and the anterior ethmoidal artery was cauterized to achieve hemostasis. The tumor displaced the skull base, but there was no evidence of cerebrospinal fluid (CSF) leakage. Small remnants in direct contact with the skull base were left in situ to avoid iatrogenic injuries (Figure 1).

**Figure 2:**
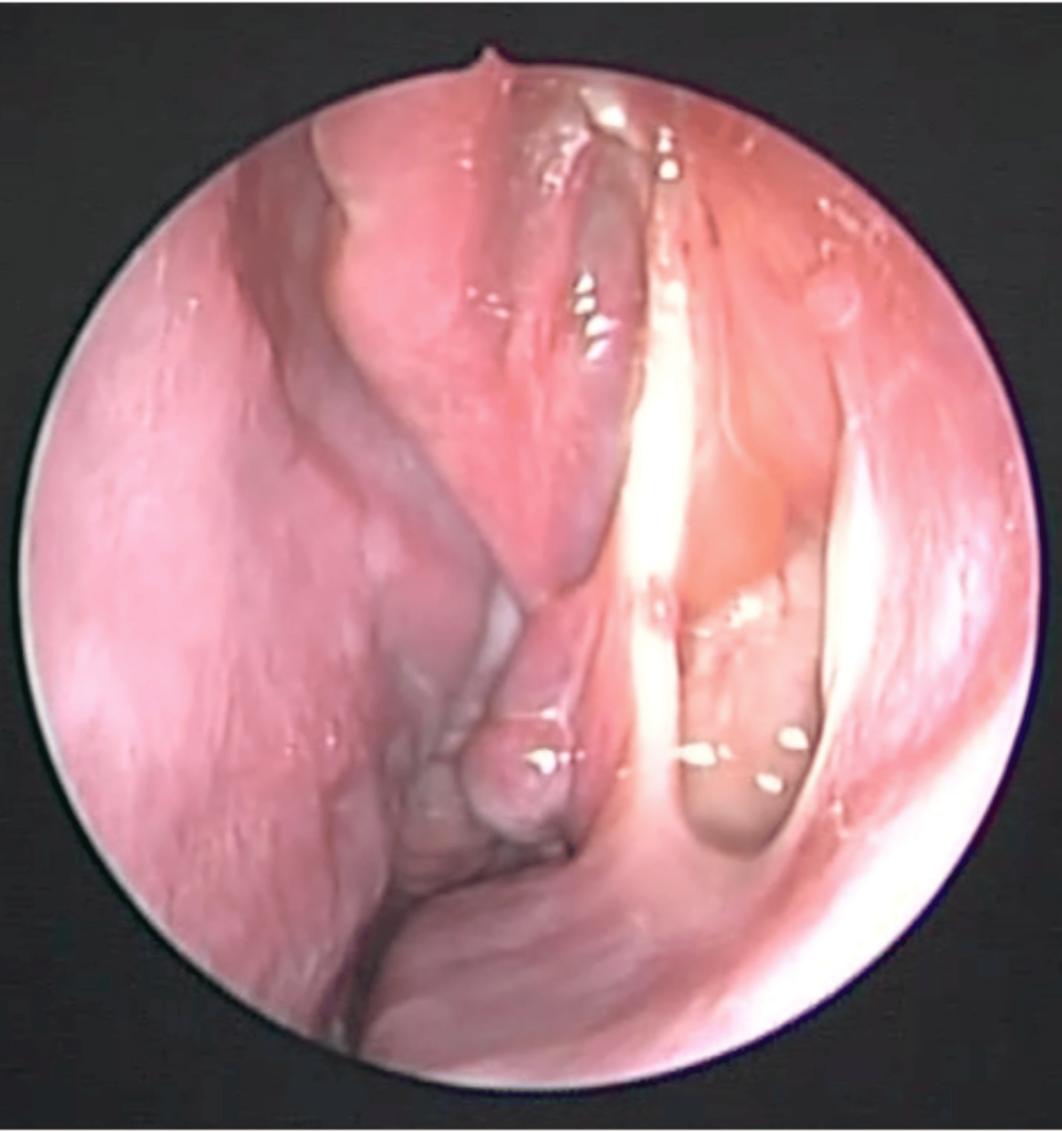
Case 1: Nasal endoscopy at the six-month follow-up Left nasal cavity with maxillary antrostomy and ethmoidectomy without residual tumor.

The operative time was approximately three hours, with an estimated blood loss of 500 ml. The patient required transfusion of one unit of blood products, and norepinephrine was administered intraoperatively because of transient hypotension. Postoperatively, the patient developed severe bronchospasm following extubation, with oxygen saturation dropping to 69-79%. After airway clearance and supportive management, the ventilation and oxygenation normalized. The patient was transferred to the Pediatric Intensive Care Unit (PICU) for continued observation and postoperative care, where he remained intubated for 12 hours for respiratory stabilization and was subsequently extubated uneventfully. Postoperative CT revealed no residual lesions or skull base complications. The patient was discharged on postoperative day two without complications. The definitive histopathological result was a JOF psammomatoid variant. At the six-month follow-up, the patient was asymptomatic, and nasal endoscopy showed no evidence of recurrence (Figure 2).

Case 2

**Figure 3:**
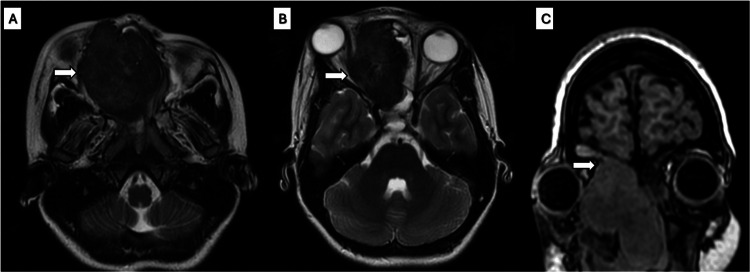
Case 2: Preoperative MRI A) Axial T1-weighted image demonstrating a heterogeneous expansile mass in the right nasal cavity displacing the right orbit laterally (white arrow). B) Axial T2-weighted image showing orbital involvement (white arrow). C) Coronal T1-weighted image demonstrating the involvement of the anterior cranial fossa (white arrow).

A previously healthy 13-year-old female initially presented with intermittent bilateral nasal congestion and occasional epistaxis. One month later, she developed recurrent right-sided epistaxis and progressive swelling of the right orbital region. Thyroid function was normal; however, CT imaging revealed a right nasal mass, prompting a referral to a tertiary care center. A biopsy of the right nasal fossa was initially interpreted as monostotic fibrous dysplasia. Given the unusual presentation, an oncologist requested a pathology review, which revised the diagnosis to an ossifying fibroma. Subsequent MRI demonstrated an expansile lesion involving the right nasal cavity and paranasal sinuses, extending superiorly into the anterior cranial fossa (Figure 3).

**Figure 4:**
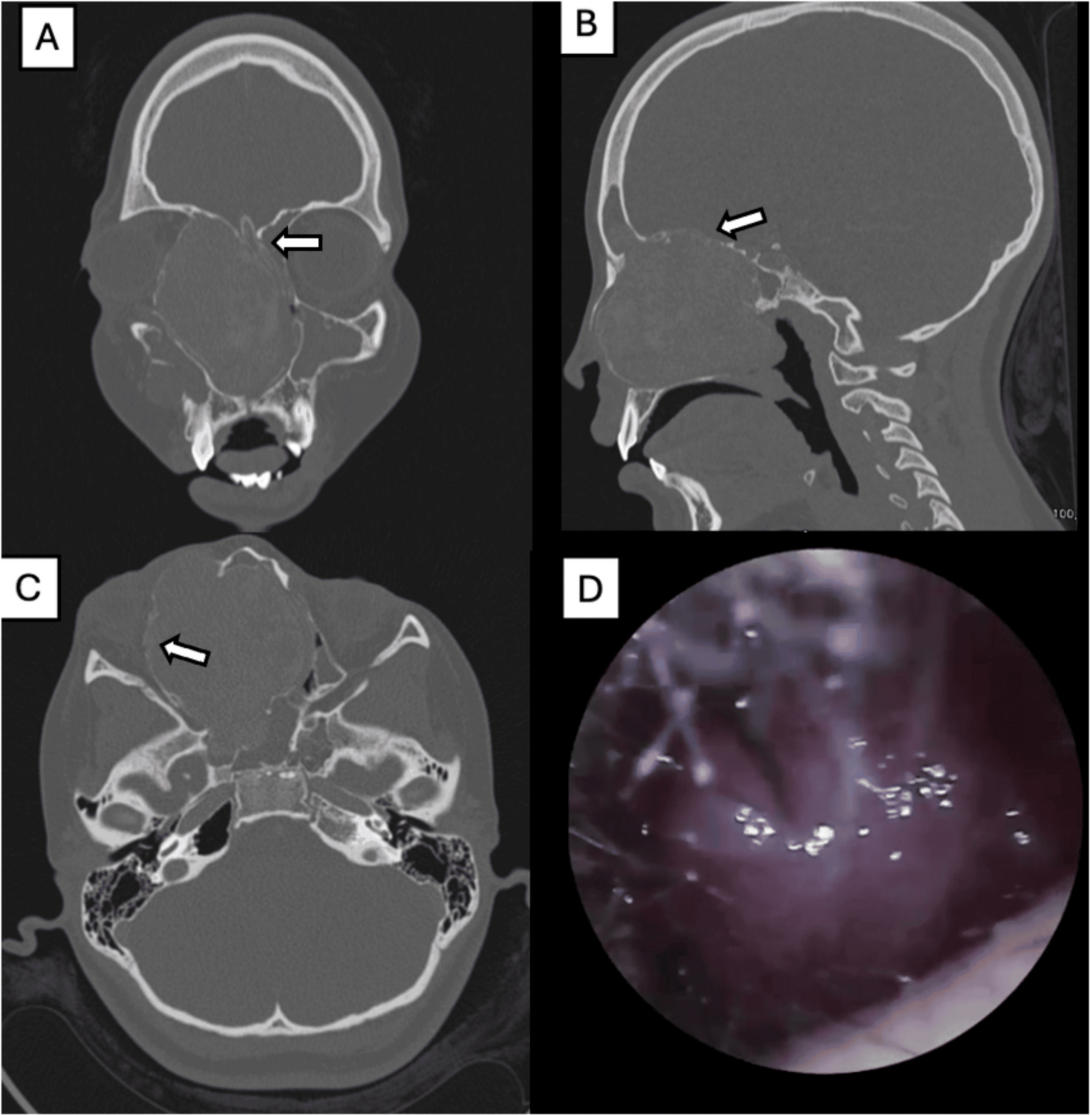
Case 2: Preoperative simple CT scan and nasal endoscopy. A) Coronal view: expansile soft-tissue density lesion occupying the right nasal cavity, with extension into the ipsilateral orbit and thinning of the adjacent bony walls (white arrow). B) Sagittal view: anterior right nasal cavity to the nasopharynx and superiorly into the right frontal sinus, sphenoid sinus, and anterior cranial fossa. C) Axial view: The mass displaced and remodeled the medial wall of the right orbit (white arrow), elongated the optic nerve, and caused ipsilateral proptosis. D) A well-defined pink, rounded, smooth mass with focal hemorrhagic areas arising from the right nasal cavity and extending toward the nasal vestibule.

Following symptom onset, the patient experienced a nine-month delay related to loss of follow-up due to prolonged scheduling of multidisciplinary consultations and delayed completion of diagnostic imaging studies. She subsequently presented with a 12-hour history of severe pulsatile pain in the right malar region, associated with erythema, increased local temperature, and right eyelid edema, without limitations in eye movement or visual acuity. Upon evaluation in the emergency department, the patient was found to be febrile, alert, and hemodynamically stable, with no neurological deficits observed. There was a significant increase in volume in the right nasal dorsum region, with axial and later inferior proptosis of the right eye globe, which was displaced in a disfiguring manner, with infraorbital edema and erythema. Nasal endoscopy revealed a well-defined, rounded, and smooth mass with focal hemorrhagic areas arising from the right nasal cavity and extending toward the nasal vestibule. Contrast-enhanced CT of the skull revealed an expansive, lobulated, well-defined lesion in the right nasal cavity. The lesion measured approximately 6.7 × 5.8 × 5.1 cm, with a volume of approximately 103 cc. It extended from the anterior nasal cavity to the nasopharynx and superiorly into the right frontal sinus, sphenoid sinus, and anterior cranial fossa (Figure 4).

The mass displaced and remodeled the medial wall of the right orbit, elongated the optic nerve, and caused ipsilateral proptosis. Bone erosion of the hard palate and maxillary sinus expansion with mucocele were also noted.

**Figure 5:**
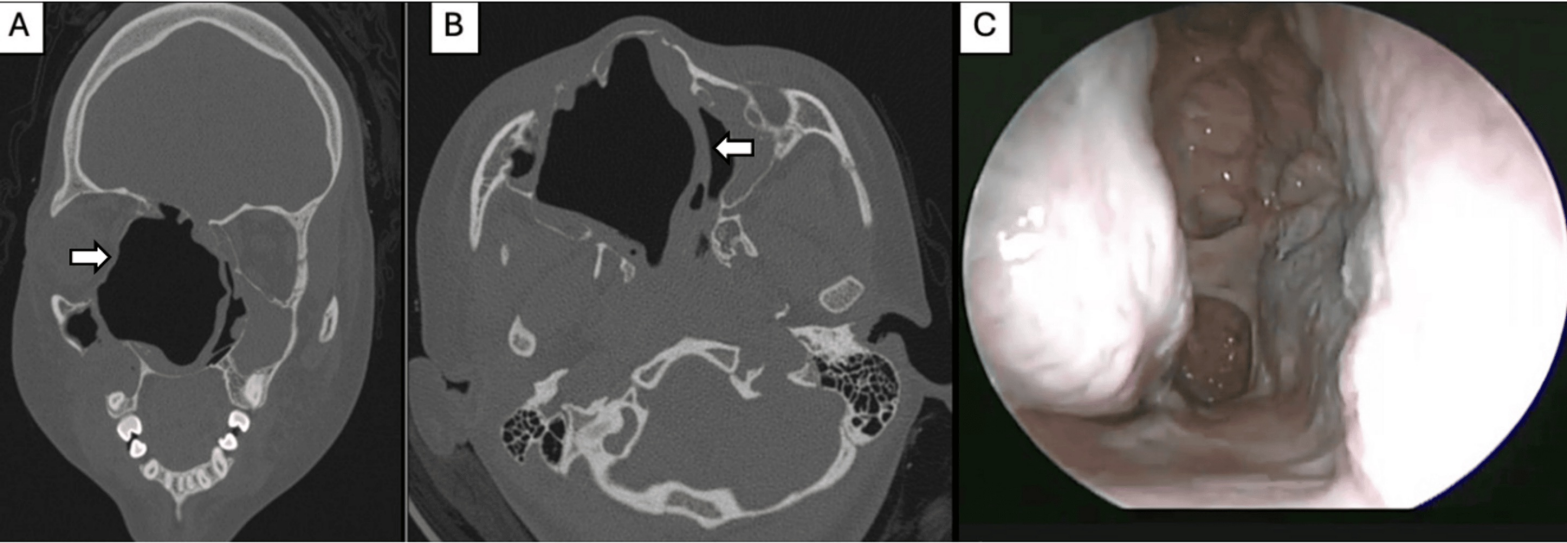
Case 2: Postoperative CT and nasal endoscopy of the right nasal cavity. A) Coronal view without tumor and orbital displacement to the right with lamina papyracea erosion (white arrow). B) Axial view of the nasal septum displaced to the left (white arrow). C) Nasal endoscopy of a patent right nasal cavity without signs of tumor recurrence up to the nasopharynx.

Despite medical management with analgesics and antibiotics, the patient’s facial swelling worsened within 12 hours after discharge. A subsequent MRI confirmed the presence of a 7.1 x 5.0 x 5.8 cm lesion extending from the anterior nasal cavity to the nasopharynx, frontal sinus, sphenoid sinus, and anterior cranial fossa. The patient was admitted for intravenous antibiotics (ceftriaxone, vancomycin, and metronidazole) and underwent functional endoscopic sinus surgery and Caldwell-Luc surgery. Intraoperatively, the patient received a transfusion due to significant blood loss (1000 ml). Postoperatively, the patient was stable and was discharged five days later without complications. The patient was prescribed trimethoprim-sulfamethoxazole for an additional week post-discharge, with scheduled follow-up appointments with both infectious disease and otorhinolaryngology services. A computed tomography (CT) scan performed five days postoperatively confirmed the presence of postsurgical changes, with no evidence of residual lesions. The histopathology report was consistent with JOF, and throughout the follow-up period, there were no clinical manifestations or endoscopic evidence consistent with disease recurrence (Figure 5).

Case 3

**Figure 6:**
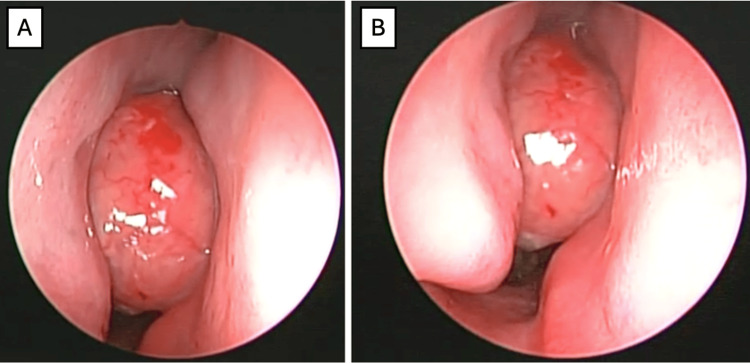
Case 3: Initial nasal endoscopy. Right nasal cavity with the presence of a regular, rounded mass protruding from the middle meatus (A) and contacting both the nasal septum and the inferior turbinate (B).

A 16-year-old male with no significant past medical history presented with a three-year history of spontaneous, recurrent, right-sided epistaxis of variable severity. Over time, he developed a painless, slowly enlarging mass in the right upper eyelid, which caused partial eyelid closure and mild diplopia on rightward gaze. He also reported right-sided nasal obstruction and intermittent epiphora without facial pain, erythema, or conjunctival injection. Upon examination, the patient was alert, cooperative, and neurologically intact with no signs of respiratory distress. Notable findings included a palpable soft mass in the right eyelid, which caused limited eyelid opening and mild diplopia on rightward gaze. Nasal endoscopy was performed with evidence of a rounded, regular mass in the right nasal cavity (Figure 6).

Contrast-enhanced MRI revealed a solid-appearing mass with an apparent epicenter in the right nasal cavity, occupying and expanding the entire anterior osteomeatal complex. Hypointensity on T2 and T1 sequences, with heterogeneous post-gadolinium enhancement, presented a central bone matrix, extending and invading the right frontal and maxillary sinuses, with apparent erosion of the posterior margin of the ipsilateral frontal sinus. It extended into the right extraconal space through dehiscence of the ipsilateral lamina papyracea, causing displacement of intraconal structures to the right and proptosis. These findings were suggestive of an ossifying nasosinusal fibroma.

He underwent endoscopic resection, and nasal exploration revealed a firm, pink, round mass between the inferior and middle turbinates. The tumor was dissected and partially excised using a monopolar cautery. Due to orbital involvement, a Lynch incision was made to expose and resect the tumor, followed by an endoscopic tumor resection. The tumor extended into the maxillary sinus, necessitating medial maxillectomy. Post-surgery, the patient showed no signs of active hemorrhage. Examination revealed a well-healing Lynch incision without dehiscence, inflammation, or infection. The right nasal cavity exhibited postoperative changes consistent with endoscopic tumor resection, including the absence of the middle turbinate and evidence of medial maxillectomy. Ocular movements were preserved, with mild diplopia on rightward gaze. The histopathology report was consistent with JOF, and no clinical or imaging findings suggestive of recurrence were observed during the six-month follow-up.

Table 1 presents a comparison of laboratory findings, surgical time, bleeding, and approach type for each case.

**Table 1:** Laboratoy results and Surgical parameters OR: operation room, CRP: C-reactive protein, ESR:  erythrocyte sedimentation rate

Parameter	Values			Range (Unit)
Laboratory Results	Case 1	Case 2	Case 3	
Hemoglobin	14.7	14.7	14.7	12.2-18.1 (G/dL)
Hematocrit	42.5	42.5	42.5	Men: 40-54% Women: 36-48%
Leukocytes	5.9	5.9	5.9	2.00-6.90 (K/uL)
Platalets	177	177	177	142.00-424.00 (K/uL)
CRP	10.3	10.3	10.3	0-1.00 (mg/dL)
ESR	38	38	38	0-9 (mm/hr)
Surgical parameters				
OR time	180	900	250	Minutes
Transurgical blood loss	500	1000	600	Mililiters
Mixed approach	Endoscopic	cad-well	Lynch	Endoscopic

## Discussion

Juvenile ossifying fibroma is a benign but locally aggressive fibro-osseous lesion that predominantly affects the craniofacial skeleton in the pediatric population. Its rapid expansion and tendency to involve the orbit and anterior skull base pose significant diagnostic and therapeutic challenges [1,2,5,9]. In our series, all three pediatric patients presented with sinonasal and orbital involvement, consistent with previous reports identifying the ethmoid and maxillary sinuses as the most common sites of origin, frequently associated with orbital and intracranial extension [3-5,10].

The clinical presentation of juvenile ossifying fibroma (JOF) is often subtle and progressive, which may delay diagnosis and treatment. Common manifestations include facial swelling, nasal obstruction, proptosis, and epistaxis. In our patients, the initial symptoms consisted primarily of recurrent epistaxis and progressive proptosis, findings that align with those reported in the literature [3,5]. These nonspecific manifestations may mimic inflammatory or vascular lesions, underscoring the importance of early imaging and histopathological evaluation to establish an accurate diagnosis [7,8].

Radiologic evaluation plays a central role in differentiating JOF from other fibro-osseous or neoplastic entities. On CT imaging, these lesions typically appear as well-circumscribed expansile masses with a thin sclerotic rim and central lytic component, often described as having an “eggshell” appearance. MRI usually demonstrates low-to-intermediate signal intensity on T1-weighted sequences and variable T2 signal intensity, with enhancement of the outer rim following contrast administration [6,12]. These imaging characteristics were consistently observed across all three cases in our series.

Histopathologically, juvenile trabecular ossifying fibroma and juvenile psammomatoid ossifying fibroma represent distinct variants, although overlapping clinical behavior has been described despite their differing microscopic features [6]. The psammomatoid subtype more frequently involves the paranasal sinuses and orbit, whereas the trabecular variant predominantly affects the maxilla [2,11-13]. All cases in our series occurred within the pediatric age range typical of JOF and demonstrated variable histologic subtypes, highlighting the heterogeneity of this entity.

Surgical excision remains the primary treatment for JOF. While complete resection is generally recommended due to the lesion’s locally aggressive nature and risk of recurrence [6,11,13], the extent of surgery must be carefully balanced against the risk of morbidity. Historically, en bloc resection was considered the gold standard for aggressive lesions; however, recurrence rates of up to 30% have been reported following conservative approaches, particularly when margins are incomplete or lesions are adjacent to vital structures [2-4,7,11]. Current practice supports individualized surgical planning that prioritizes complete excision when safely achievable while preserving function and aesthetics [2,5,9,10].

Advances in endonasal endoscopic techniques have significantly improved the management of fibro-osseous lesions of the paranasal sinuses by enhancing visualization, reducing morbidity, and providing favorable cosmetic outcomes [5,7]. Nevertheless, combined external and endoscopic approaches may be required for large lesions with orbital, maxillary, or skull base extension when complete endonasal resection is not feasible. In such cases, recurrence has been reported to be uncommon when adequate resection is achieved, although long-term follow-up remains essential [5,6].

In our series, endoscopic resection was the primary surgical approach. Two patients required a combined approach: one underwent a Caldwell-Luc procedure for maxillary extension, and the other required a Lynch incision to access the orbital component. In one case, small tumor remnants were intentionally left in situ due to close proximity to the skull base, as aggressive resection would have posed an unacceptable risk of neurological or cerebrospinal fluid-related complications. In these selected scenarios, preservation of function may outweigh the benefits of radical excision, particularly in pediatric patients, and close clinical and radiologic surveillance is considered an appropriate management strategy. Adjuvant therapy is not routinely indicated for JOF [6,11,13].

Significant intraoperative blood loss represents a recognized challenge during resection of JOF, reflecting both the intrinsic vascularity of the lesion and the highly vascular pediatric craniofacial anatomy [1,3,6]. Lesions involving the sinonasal cavity, orbit, or anterior skull base are particularly prone to substantial bleeding [2,7-9]. Transcatheter arterial embolization (TAE) management of sinonasal tumors in pediatrics has been widely described forvascular etiology tumors such as juvenile nasopharyngeal angiofibroma (JNA), as well as for the management of other highly vascularized tumors, such as pyogenic granuloma and nasal polyps [14-16]. Accordingly, meticulous preoperative planning is essential and may include detailed vascular assessment on contrast-enhanced imaging and, in selected cases, consideration of preoperative embolization for large or hypervascular lesions [1,10]. Intraoperative strategies to mitigate blood loss include careful surgical technique, staged or combined approaches, early control of feeding vessels, hypotensive anesthesia, and readiness for blood product transfusion [6,8,11,13]. Although intraoperative bleeding occurred in two cases in our series, all patients recovered uneventfully without visual or neurological deficits.

Recurrence remains a major concern in JOF, with reported rates ranging from 20% to 30%, particularly following incomplete resection [2,15]. While no recurrence was observed during our follow-up period (range: 3-6 months), this reflects short-term outcomes only and is insufficient to definitively assess recurrence risk in a lesion known for late recurrence. Long-term surveillance with periodic imaging is essential, as delayed recurrences have been documented years after initial treatment [2,10,12]. Our institutional protocol includes clinical follow-up at 6-month intervals for the first two years, with radiological assessment if needed, followed by annual imaging for at least five years, in accordance with the current recommendations for fibro-osseous lesions [17].

## Conclusions

Juvenile ossifying fibroma is a rare, benign, yet locally aggressive fibro-osseous lesion that can involve the orbit and anterior skull base, making early diagnosis essential. Recognition through imaging and histopathological confirmation allows for appropriate management, reducing morbidity and the risk of complications. Surgical excision remains the cornerstone of the treatment. Endoscopic approaches, either alone or in combination with external techniques, provide safe and effective tumor removal with minimal morbidity and excellent functional and cosmetic outcomes. However, due to the potential for recurrence, long-term follow-up and multidisciplinary collaboration among radiologists, pathologists, and surgeons are crucial to ensure successful outcomes and minimize disease relapse.
